# Right Congenital Diaphragmatic Hernia Associated With Hepatic Pulmonary Fusion: A Case Report

**DOI:** 10.21699/jns.v5i3.370

**Published:** 2016-07-03

**Authors:** Rachida Laamiri, Samia Belhassen, Amine Ksia, Amina Ben Salem, Nahla Kechiche, Sana Mosbahi, Lassaad Sahnoun, Mongi Mekki, Mohsen Belghith, Abdellatif Nouri

**Affiliations:** 1 Department of Pediatric Surgery, Fattouma Bourguiba University Hospital- Monastir, Tunisia; 2 Department of Radiology B, Maternal and Fetal Unit, Fattouma Bourguiba University Hospital-Monastir, Tunisia

**Keywords:** Congenital diaphragmatic hernia, Hepatic pulmonary fusion, Respiratory distress

## Abstract

We present a case of male newborn presented with respiratory distress at 21 hours of life. The patient was operated for right congenital diaphragmatic hernia (CDH). Hepatic pulmonary fusion (HPF) was found at surgery.

## CASE REPORT

A 21-hour old newborn male, born at a gestational age of 41 weeks and weighing 3.14 kg, presented with respiratory distress. Chest radiograph showed right diaphragmatic hernia and complete opacification of the right hemithorax with displacement of mediastinal structure to the left hemithorax (Fig. 1). The patient was intubated and put on oscillatory mechanical ventilation; dobutamine and dopamine were also started. After initial stabilization, the patient underwent repair of right CDH via a right supra-umbilical transverse incision. At operation, there was an 8cm wide posterolateral defect of the right hemidiaphragm, with the liver herniated into and filling most of the right hemithorax. The diaphragmatic defect was enlarged to the liver was delivered back to the abdomen. During reduction, it was noted that the severely hypoplastic right lung was fused to the dome of the liver (Fig. 2). No distinct plane between the liver and the lung could be established while dissection. The patient developed severe bradycardia during surgery; therefore, decision was made to partially fermeture the diaphragmatic defect. We closed the vast majority of the diaphragmatic defect postero-laterally, leaving enough space medially for the fused lung and liver. Postoperative course remained stormy and patient succumbed to progressive pulmonary failure.

**Figure:**
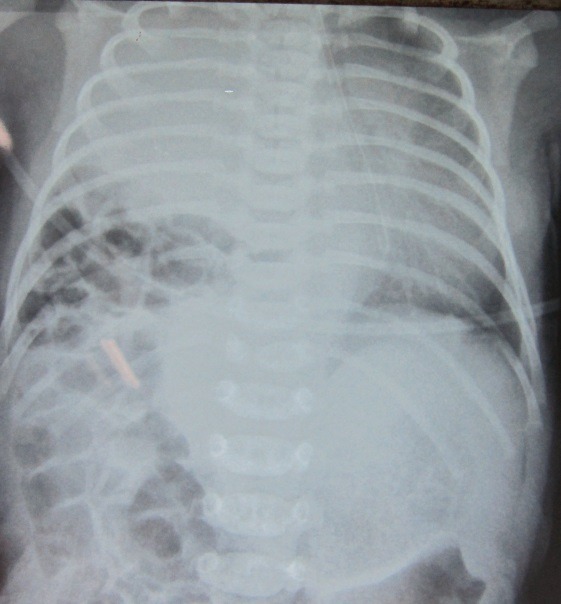
Figure 1: Showing right CDH, and mediastinal shift.

**Figure:**
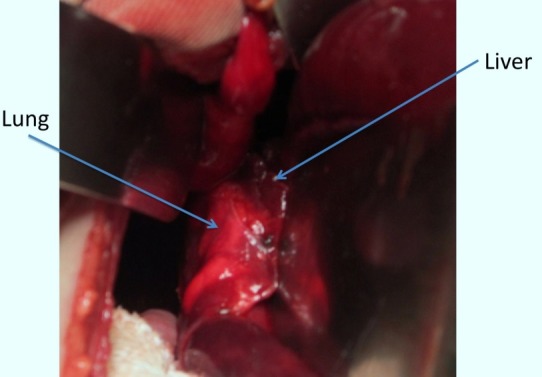
Figure 2: Showing HPF.

## DISCUSSION

HPF is an extremely rare condition in patients with congenital diaphragmatic hernia and only 14 cases have been reported in literature.[1-3] Most of these cases presented in the early neonatal period or infancy. There may be varying degrees of pulmonary hypoplasia associated with this condition.[4] The clinical presentations of HPF is not different to that of congenital diaphragmatic hernia without HPF.[1] The common radiological finding in case of HPF is the opacification of the right hemithorax with or without mediastinal shift to the contralateral hemithorax or toward the lesion.[4] In the index case, radiograph showed opacification of right chest with mediastinal shift to the contralateral hemithorax. Hepatic and pulmonary tissues fuse by a fibrous band and broncho-biliary fistula formation are the other reported findings associated with HPF.[2] Anomalous venous drainage from the right lung to the intrahepatic inferior vena cava was also reported.[1,5]

Treatment of congenital diaphragmatic hernia with HPF is surgical repair, which may require partial hepatectomy and/or pneumonectomy.[6] The separation between the two organs can be challenging and difficult to perform. Robertson et al, [2] used LigaSure device (Valley Lab, Boulder, Colo) in order to separate the lung from the liver, and in another case, attempted blunt dissection created a large air leak.[2,4] Slovis et al [4] described 2 cases in whom the defect was repaired without separation of the fused tissue. In the case reported by Gander [7], the defect was approximated around the HPF. And they chose to use polyester fiber mesh on the liver capsule with the hope that this would result in an inflammatory reaction that might incorporate the patch. The prognosis of patients with HPF is poor. Most patients usually die during the perioperative period due to various complications, including respiratory distress, right heart failure, persistent pulmonary hypertension, and thrombosis of inferior vena cava.[2,6] There are 8 deaths among 14 reported cases of HPF.[2,3] The poor prognosis is usually attributed to CDH related pulmonary complications and any further negative impact on prognosis by the presence of HPF is not proven as yet.

## Footnotes

**Source of Support:** Nil

**Conflict of Interest:** Nil
